# Association of Matrix Metalloproteinase-3 -1171(5A>6A) Polymorphism with Cancer Risk: A Meta-Analysis of 41 Studies

**DOI:** 10.1371/journal.pone.0087562

**Published:** 2014-01-29

**Authors:** Xin Yang, Jing-Wen Hu, Man-Tang Qiu, Ming Li, Rong Yin, Jie Wang, Lin Xu, Qin Zhang

**Affiliations:** 1 The First Clinical College of Nanjing Medical University, Nanjing, China; 2 Department of Thoracic Surgery, Nanjing Medical University Affiliated Cancer Hospital, Cancer Institute of Jiangsu Province, Nanjing, China; 3 Jiangsu Key Laboratory of Molecular and Translational Cancer Research, Nanjing, China; 4 Department of Scientific Research, Nanjing Medical University Affiliated Cancer Hospital Cancer Institute of Jiangsu Province, Nanjing, China; State University of Maringá/Universidade Estadual de Maringá, Brazil

## Abstract

**Background and Objective:**

Evidence has shown that matrix metalloproteinases-3 (MMP3) is important for cancer progression. Recent studies about the association between the -1171(5A>6A) polymorphism in MMP3 promoter region and cancer risk have yielded conflicting results.

**Methodology/Principal Findings:**

We performed a meta-analysis of 41 studies including 11112 cases and 11091 controls to determine whether the -1171(5A>6A) polymorphism of MMP3 was associated with cancer risk. We assessed the strength of association and performed sub-group analyses by cancer types, ethnicity, smoking status, genotyping method, source of controls and sample size. The pooled results revealed that no significant association of the -1171(5A>6A) polymorphism with overall cancer risk in any of four models. Further sub-group analysis revealed that individuals with the 6A allele had lower risk of gastrointestinal cancer in two models: heterozygote comparison (6A/5A vs. 5A/5A: OR = 0.74, 95%CI: 0.60—0.91; I^2^ = 1.9%), and dominant model (6A/6A+6A/5A vs. 5A/5A: OR = 0.77, 95%CI: 0.64—0.94; I^2^ = 29.0%). Additionally, the associations were significant in Asian populations for three models: homozygote comparison (6A/6A vs. 5A/5A, OR = 0.68, 95%CI: 0.52—0.90; I^2^ = 26.7%), heterozygote comparison (6A/5A vs. 5A/5A: OR = 0.75, 95%CI: 0.58—0.98; I^2^ = 0.0%), and dominant model (6A/6A+6A/5A vs. 5A/5A: OR = 0.69, 95%CI: 0.54—0.88; I^2^ = 0.5%). It was noteworthy that we had a contrary finding in non-smokers: the variant 6A/6A homozygote might statistically increase cancer risk compared with 6A/5A+5A/5A genotype (OR = 1.92, 95%CI: 1.25—2.96; I^2^ = 72.7%).

**Conclusion:**

This meta-analysis suggests that the -1171(5A>6A) polymorphism in MMP3 promoter region is not associated with overall cancer risk, but it may contribute to decreased cancer risk in Asian population when compared with Caucasian population and significantly reduce the risk of gastrointestinal cancer.

## Introduction

The matrix metalloproteinases (MMPs), a family of highly conserved zinc-dependent proteolytic enzymes that degrade many different components of the extracellular matrix (ECM) and basement membrane, have been involved in the regulation of various cell behaviors with relevance to tumor development and metastasis [1]–[3]. MMPs are divided into five subgroups according to their structure and substrate specificity: collagenases, stromelysins, gelatinases, membrane-type MMPs, and other MMPs [4]. MMPs are classified into 24 enzymes according to substrate specificity and structural similarities [5]. Expression of most MMPs in tumors is regulated primarily at the transcriptional level, but there is also evidence of modulation of mRNA stability in response to growth factors and cytokines secreted by tumor-infiltrating inflammatory cells as well as by tumor and stromal cells [6].

MMP3 (stromelysin-1) is known to lyse basal membrane collagen and induce the synthesis of other MMPs such as MMP1 and MMP9 [7], [8]. The MMP3 gene is localized on 11q22 adjacent to the MMP1 gene, produced by stromal fibroblasts, macrophages and synovial cells [9]. A single adenine insertion/deletion polymorphism (5A>6A) at the 1171 position of the MMP3 promoter region could modulate its transcription [10]. *In vitro* assays of promoter activity showed that the 5A allele had a two-fold higher promoter activity than the 6A allele [10]. A large number of studies have demonstrated the association between MMP3 -1171(5A>6A) polymorphism and cancer risk, including colorectal, lung, head and neck, esophagus, breast, ovarian cancers and so on [6], [11]–[15]. However, these studies yielded different or even controversial results. For example, Ghilardi et al. [11] found a significant association between MMP3 -1171 5A allele and increased cancer risk, but Su et al. [15] reported no significant correlation.

Meta-analysis is a means of increasing the effective sample size through pooling of data from individual studies, thus enhancing the statistical power of the analysis for the estimation of genetic effects [16]. To clarify the association between MMP3 -1171(5A>6A) polymorphism and cancer risk, we performed this meta-analysis by pooling eligible studies to calculate the estimate of overall cancer risk and evaluated influence of cancer types, ethnicity, smoking status, genotyping method, source of controls and sample size.

## Methods

### Literature Search Strategy and Selection Criteria

This meta-analysis was designed, conducted, and reported according to the PRISMA guideline [17]. We carried out literature search in the PubMed, EMBASE and CNKI (Chinese National Knowledge Infrastructure) without language, time period and sample size limitations, covering all papers published up to August 21, 2013, with a combination of the following keywords: MMP3 gene (e.g.: ‘‘MMP3’’, or ‘‘matrix metalloproteinase-3’’); cancer (e.g.: ‘‘cancer’’, ‘‘carcinoma’’, ‘‘tumor’’ or ‘‘neoplasms’’) and polymorphism or variation. Before searching Pubmed database, we searched MeSH database to find the most matched searching items. And for the descriptor “polymorphism”, we used MeSH word “Polymorphism, Single Nucleotide” in the searching strategy. In addition, we performed manual search of references of relative articles and reviews. The following criteria was used for the literature selection: (a) case–control studies or cohort studies; (b) investigating the association between the -1171(5A>6A) polymorphism in MMP3 promoter region and cancer risk; (c) sufficient genotype distribution information in cases and controls. The major reasons for exclusion of studies were (a) reviews and duplicated reports from the same study; (b) study design other than case-control method; (c) studies without detailed genotype frequencies.

### Data Extraction

Data were extracted from all eligible publications independently by two of the authors (Yang and Hu) according to the selection criteria from each of the eligible papers: name of first author, publication year, country where the study was conducted, ethnicity, source of controls, cancer types and genotyping methods, total number of cases and controls, genotype frequency in cases and controls. Different ethnicities were categorized as Asian and Caucasian. Cancer types were classified as Gynecological cancer (GC), including ovarian, cervical and endometrial cancer; Gastrointestinal cancer (GIC), including gastric and colorectal cancer; Breast cancer (BC); Head and neck cancer (HNC); Hepatocellular carcinoma(HC); Lung cancer(LC); Oral cancer(OC); Others (renal cell carcinoma, esophageal cancer, bladder cancer, brain astrocytoma and nasopharyngeal carcinoma). All eligible studies were defined as hospital-based(HB), population-based(PB), friends and spouse-based(FASB) according to the source of controls. The Hardy–Weinberg equilibrium (HWE) was calculated by Chi-square test (p<0.05 was considered as significant disequilibrium) based on -1171 5A>6A polymorphism genotyping distribution in controls [18].

### Statistical Analysis

The strength of the association between MMP3 -1171(5A>6A) polymorphism and cancer risk was estimated by calculating odds ratio (OR) with 95% confidence intervals (95% CI), based on the genotype frequencies in cases and controls. The pooled ORs were calculated for four models: homozygote comparison (6A/6A vs. 5A/5A), heterozygote comparison (6A/5A vs. 5A/5A), dominant model (6A/6A+6A/5A vs. 5A/5A) and recessive model (6A/6A vs. 6A/5A+5A/5A). The fixed effects model (Mantel-Haenszel method) was used when there was no significant heterogeneity [19]; otherwise, the random effects model (the Der Simonian and Laird method) was utilized [20]. According to the Cochrane Handbook for Systematic Reviews of Interventions, a useful statistic for quantifying inconsistency is I  =  [(Q –df)/Q]×100%, where Q is the chi-squared statistic and df is its degrees of freedom. This describes the percentage of the variability in effect estimates that is due to heterogeneity rather than sampling error. The value of I^2^>50% indicates substantial heterogeneity. Sensitivity analysis was conducted by deleting each individual study in turn from the total and reanalyzing the remainder [21]. Sub-group analyses and logistic meta-regression analyses were conducted to explore the source of heterogeneity among variables, such as cancer types, ethnicity, genotyping method, source of controls and sample size (studies with more than 1000 participants were defined as ‘‘large’’, and studies with less 1000 participants were defined as ‘‘small’’). Publication bias was both examined with Begg’s funnel plot [22] and Egger’s regression method [23] (p<0.05 was considered representative of statistically significant publication bias). All p values are two-sided. Data were analyzed using STATA software (version 12.1; Stata Corp, College Station, Texas USA).

## Results

### Characteristics of Studies

**Figure 1:**
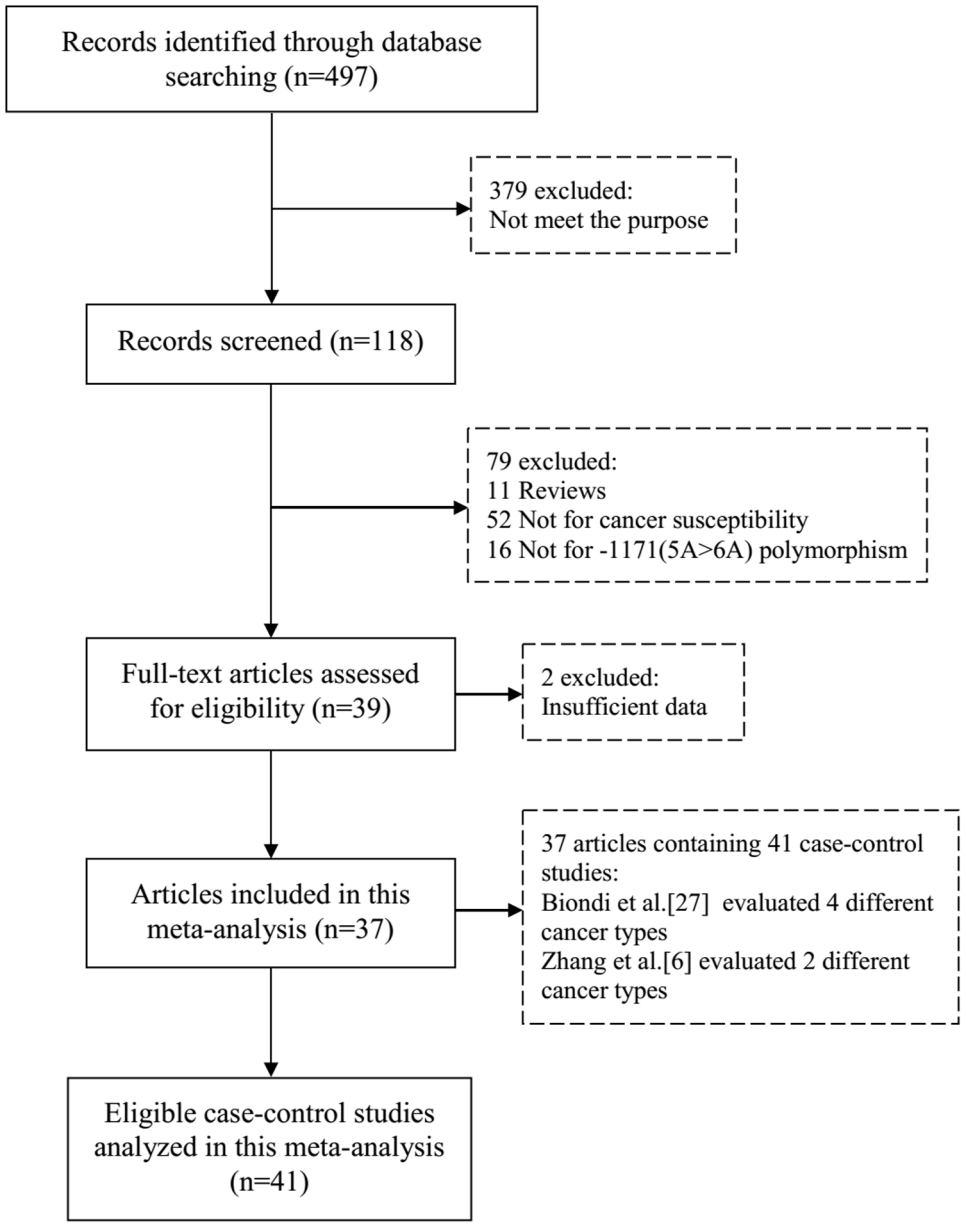
PRISMA Flow Chart.

The detailed screening process was shown in Figure 1. Finally, there are a total of 41 eligible case-control studies included in this meta-analysis, containing 11112 cases and 11091 controls [6], [9], [11]–[15], [24]–[53]. In the study reported by Biondi et al. [27], the cancer types contained breast, colorectal, ovarian and lung cancer; and in another study reported by Zhang et al. [6], the cancer types included esophageal and gastric cancer. And the genotype frequencies were presented separately, thus each of them was considered as a separate study in this meta-analysis. There were 23 studies conducted in Asians, and 18 studies conducted in Caucasians. Population-based controls were used in 15 studies and hospital-based controls were used in 24 studies. There were 7 studies of large sample size and 34 studies of small sample size. The detailed characteristics of the eligible studies included in this meta-analysis are shown in Table 1.

**Table 1:** Characteristics of Eligible Studies.

First author	Year	Ethnicity	Cancer types	Control	Genotyping method	Cases			Controls			HWE
						5A/5A	5A/6A	6A/6A	5A/5A	5A/6A	6A/6A	
**Biondi ** [**27**]	2000	Caucasian	Breast cancer	PB	AS-PCR	15	22	6	42	74	48	0.22
**Biondi ** [**27**]	2000	Caucasian	Colorectal cancer	PB	AS-PCR	11	35	17	42	74	48	0.22
**Biondi ** [**27**]	2000	Caucasian	Ovarian cancer	PB	AS-PCR	3	19	3	42	74	48	0.22
**Biondi ** [**27**]	2000	Caucasian	Lung cancer	PB	AS-PCR	9	14	6	42	74	48	0.22
**Lei ** [**26**]	2002	Caucasian	Breast cancer	Mixed	AS-PCR	58	127	61	47	92	43	0.88
**Ghilardi ** [**11**]	2002	Caucasian	Breast cancer	PB	AS-PCR	24	47	15	22	54	34	0.95
**Hinoda ** [**12**]	2002	Asian	Colorectal cancer	HB	PCR-RFLP	3	19	79	3	44	80	0.28
**Smolarz ** [**13**]	2003	Caucasian	Ovarian cancer	PB	AS-PCR	37	46	35	26	52	32	0.59
**Hirata ** [**24**]	2004	Asian	Renal cell carcinoma	HB	PCR-RFLP	3	38	115	4	67	159	0.31
**Hashimoto ** [**25**]	2004	Asian	Head and neck cancer	HB	PCR-RFLP	3	30	107	5	63	155	0.63
**Zinzindohoue ** [**14**]	2004	Caucasian	Head and neck cancer	HB	AS-PCR	36	70	19	60	121	68	0.67
**Krippl ** [**28**]	2004	Caucasian	Breast cancer	PB	Taqman	103	259	138	115	233	145	0.26
**Zhang ** [**6**]	2004	Asian	Esophageal cancer	HB	PCR-RFLP	1	73	160	8	105	237	0.36
**Zhang ** [**6**]	2004	Asian	Gastric cancer	HB	PCR-RFLP	5	42	136	8	105	237	0.36
**Okamoto ** [**33**]	2005	Asian	Hepatocellular carcinoma	HB	PCR-RFLP	NA	NA	60	NA	NA	137	NA
**Fang ** [**9**]	2005	Asian	Lung cancer	HB	PCR-RFLP	7	73	163	8	105	237	0.36
**Li ** [**34**]	2005	Asian	Ovarian cancer	PB	PCR-RFLP	4	53	94	4	34	84	0.81
**Kader ** [**31**]	2006	Caucasian	Bladder cancer	HB	Taqman	134	285	136	136	277	143	0.94
**Elander ** [**30**]	2006	Caucasian	Colorectal cancer	PB	PCR-RFLP	37	52	38	48	115	45	0.13
**Su ** [**15**]	2006	Caucasian	Lung cancer	FASB	Taqman	485	1012	517	325	648	350	0.47
**Tu ** [**42**]	2006	Asian	Oral cancer	HB	AS-PCR	0	31	119	1	12	85	0.45
**Lievre ** [**29**]	2006	Caucasian	Colorectal cancer	HB	AS-PCR	158	271	166	130	291	126	0.13
**Li ** [**39**]	2006	Asian	Ovarian cancer	HB	PCR-RFLP	4	34	84	4	53	94	0.28
**Xu ** [**32**]	2006	Asian	Colorectal cancer	HB	PCR-RFLP	1	23	102	1	27	98	0.56
**Lu ** [**43**]	2007	Asian	Brain astrocytoma	HB	PCR-RFLP	5	71	145	8	109	249	0.32
**Vairaktaris ** [**35**]	2007	Caucasian	Oral cancer	PB	PCR-RFLP	36	40	84	30	51	75	<0.01
**Woo ** [**36**]	2007	Asian	Colorectal cancer	PB	PCR-RFLP	5	52	128	4	69	231	0.65
**Zhou ** [**37**]	2007	Asian	Nasopharyngeal carcinoma	PB	AS-PCR	8	149	635	5	154	604	0.15
**Lei ** [**40**]	2007	Caucasian	Breast cancer	PB	Taqman	203	478	273	206	478	262	0.66
**Zhai ** [**38**]	2007	Asian	Hepatocellular carcinoma	HB	AS-PCR	8	64	360	3	77	399	0.73
**Nishizawa ** [**41**]	2007	Asian	Oral cancer	HB	Taqman	3	50	117	8	54	102	0.81
**Han ** [**51**]	2008	Asian	Cervical cancer	HB	AS-PCR	1	16	43	3	35	62	0.46
**Vairaktaris ** [**45**]	2009	Caucasian	Oral cancer	PB	PCR-RFLP	36	84	40	30	75	51	0.80
**Okamoto ** [**50**]	2010	Asian	Hepatocellular carcinoma	HB	PCR-RFLP	3	29	60	4	27	55	0.77
**Yi ** [**47**]	2010	Asian	Endometrial cancer	HB	PCR-RFLP	4	35	79	6	51	172	0.35
**Chaudhary ** [**49**]	2010	Asian	Head and neck cancer	HB	PCR-RFLP	6	23	106	2	14	110	0.07
**Fakhoury ** [**44**]	2012	Asian	Lung cancer	PB	PCR-RFLP	26	15	0	20	24	7	0.96
**Gonzalez-Arriaga ** [**46**]	2012	Caucasian	Lung cancer	HB	PCR-RFLP	164	367	185	119	276	139	0.42
**Dey ** [**48**]	2012	Asian	Gastric cancer	HB	PCR-RFLP	16	70	132	7	38	130	0.06
**Motovali-Bashi ** [**53**]	2012	Asian	Colorectal cancer	HB	PCR-RFLP	54	55	11	24	50	26	1.00
**Grudny ** [**52**]	2013	Caucasian	Lung cancer	HB	PCR-RFLP	16	19	18	9	36	9	0.01

PB: population-based; HB: hospital-based; FASB: friends and spouse-based; HWE: Hardy–Weinberg equilibrium.

### Association between -1171(5A>6A) polymorphism and Overall Cancers Risk

As shown in Table 2, we found no significant association of the -1171(5A>6A) polymorphism in MMP3 promoter region with overall cancer risk in any of four models.

**Table 2:** Meta-analysis Results.

			6A/6A vs. 5A/5A		6A/5A vs. 5A/5A			6A/6A+6A/5A vs. 5A/5A		6A/6A vs. 6A/5A+5A/5A	
		N	OR	I^2^	OR		I^2^	OR	I^2^	OR	I^2^
**Total**		41	0.92(0.84, 1.01)	23.7%	0.95(0.87, 1.03)		14.4%	0.94(0.87, 1.01)	14.2%	0.94(0.85, 1.04)	56.2%
**Cancer Types**											
**GIC**	9		0.86(0.68, 1.09)	57.3%	**0.74(0.60, 0.91)** *		1.9%	**0.77(0.64, 0.94)** *	29.0%	0.99(0.70, 1.38)	77.9%
**GC**	6		0.86(0.53, 1.39)	0.0%	1.00(0.64, 1.55)		26.2%	0.93(0.61, 1.42)	0.0%	0.92(0.66, 1.29)	46.4%
**BC**	5		0.98(0.81, 1.18)	55.3%	1.06(0.90, 1.25)		0.0%	1.03(0.88, 1.20)	9.8%	0.87(0.66, 1.13)	54.1%
**HNC**	3		**0.51(0.29, 0.88)** *	0.0%	0.91(0.57, 1.44)		0.0%	0.75(0.49, 1.16)	0.0%	0.73(0.35, 1.52)	80.3%
**HC**	3		0.61(0.24, 1.60)	49.2%	0.58(0.22, 1.55)		51.0%	0.61(0.24, 1.58)	50.3%	0.80(0.48, 1.32)	66.8%
**LC**	6		0.95(0.81, 1.11)	0.0%	0.97(0.84, 1.12)		44.1%	0.96(0.84, 1.09)	39.4%	0.99(0.80, 1.22)	39.8%
**OC**	4		0.94(0.63, 1.40)	40.1%	0.93(0.62, 1.38)		35.8%	0.94(0.65, 1.35)	25.9%	0.94(0.64, 1.38)	55.1%
**Others**	5		0.99(0.74, 1.34)	0.0%	1.05(0.81, 1.37)		0.0%	1.03(0.80, 1.32)	0.0%	1.01(0.88, 1.16)	0.0%
**Ethnicity**											
**Caucasian**		18	0.96(0.87, 1.06)	4.3%	0.97(0.89, 1.06)		33.7%	0.97(0.89, 1.05)	1.8%	0.95(0.83,1.09)	49.3%
**Asian**		23	**0.68(0.52, 0.90)** *	26.7%	**0.75(0.58, 0.98)** *		0.0%	**0.69(0.54, 0.88)** *	0.5%	0.93(0.79, 1.09)	61.8%
**Smoking status**											
**Smoker**		7	0.95(0.75, 1.19)	0.0%	0.93(0.76, 1.15)		51.1%	0.94(0.77, 1.14)	0.0%	1.51(0.78, 2.92)	90.7%
**Non-smoker**		6	1.01(0.84, 1.21)	0.0%	1.01(0.86, 1.19)		11.4%	1.01(0.87, 1.18)	0.0%	**1.92(1.25, 2.96)** *	72.7%
**Genotyping method**											
**PCR-RFLP**		23	**0.81(0.67, 0.97)** *	18.9%	**0.78(0.66, 0.92)** *		0.0%	**0.78(0.66, 0.91)** *	0.0%	0.94(0.80, 1.12)	64.4%
**AS-PCR**		13	0.83(0.68, 1.02)	31.6%	0.89(0.75, 1.06)		23.5%	0.88(0.74, 1.03)	7.4%	0.86(0.69, 1.07)	55.0%
**Taqman**		5	1.03(0.90, 1.17)	0.0%	1.07(0.95, 1.20)		0.0%	1.05(0.94, 1.17)	0.0%	0.99(0.89, 1.09)	0.0%
**Source of Controls**											
**PB**		15	0.90(0.77, 1.05)	18.8%	0.96(0.83, 1.10)	30.4%		0.93(0.82, 1.07)	24.5%	0.88(0.74, 1.04)	44.1%
**HB**		24	0.88(0.76, 1.03)	32.3%	0.87(0.76, 1.00)	0.3%		**0.87(0.76, 0.99)** *	7.2%	0.97(0.83, 1.13)	64.8%
**FASB**		1	0.99(0.81, 1.20)	NA	1.05(0.88, 1.24)	NA		1.03(0.87, 1.21)	NA	0.96(0.82, 1.12)	NA
**Mixed**		1	1.15(0.66, 1.99)	NA	1.12(0.70, 1.79)	NA		1.13(0.72, 1.76)	NA	1.07(0.68, 1.67)	NA
**Sample Size**											
**Large^a^**		7	1.01(0.90, 1.13)	0.0%	1.00(0.91, 1.11)	4.5%		1.10(0.92, 1.11)	0.0%	1.01(0.93, 1.10)	0.0%
**Small^b^**		34	**0.75(0.63, 0.88)** *	24.3%	**0.83(0.72, 0.97)** *	7.8%		**0.79(0.69, 0.91)** *	6.9%	0.90(0.77, 1.04)	61.0%

GIC: Gastrointestinal cancer; GC: Gynecological cancer; BC: Breast cancer; HNC: Head and neck cancer; HC: Hepatocellular carcinoma; LC: Lung cancer; OC: Oral cancer; PB: population-based; HB: hospital-based; FASB: friends and spouse-based ; N: number of studies included; OR: odds ratio;

* OR with statistical significance; a: studies with more than 1000 participants; b: studies with less than 1000 participants.

### Stratified Analyses

**Figure 2:**
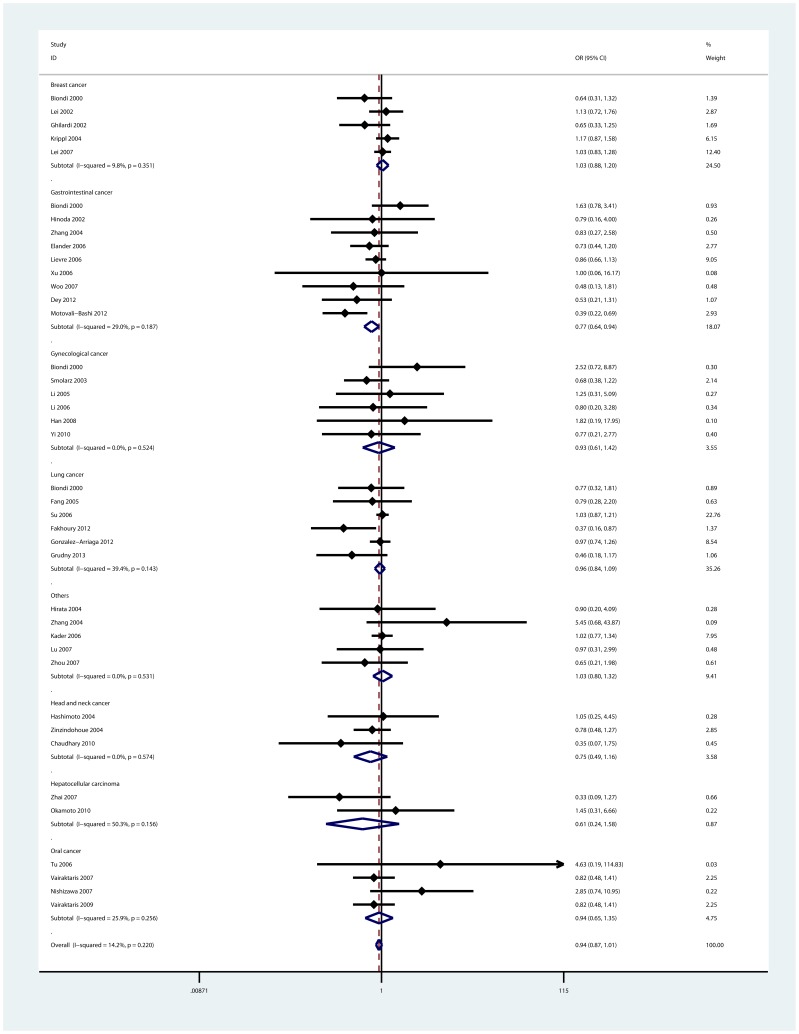
Forest plot of dominant model for overall comparison by cancer types (6A/6A+6A/5A vs. 5A/5A).

When stratified by cancer types, it was found that individuals with the 6A allele had lower risk of gastrointestinal cancer in two models: heterozygote comparison (6A/5A vs. 5A/5A: OR = 0.74, 95%CI: 0.60—0.91; I^2^ = 1.9%), and dominant model (6A/6A+6A/5A vs. 5A/5A: OR = 0.77, 95%CI: 0.64—0.94; I^2^ = 29.0%, Figure 2). In addition, we also found the -1171(5A>6A) polymorphism was associated with decreased risk of head and neck cancer in homozygote comparison (6A/6A vs. 5A/5A, OR = 0.51, 95%CI: 0.29—0.88; I^2^ = 0.0%). However, no significant association was observed for other cancer types.

**Figure 3:**
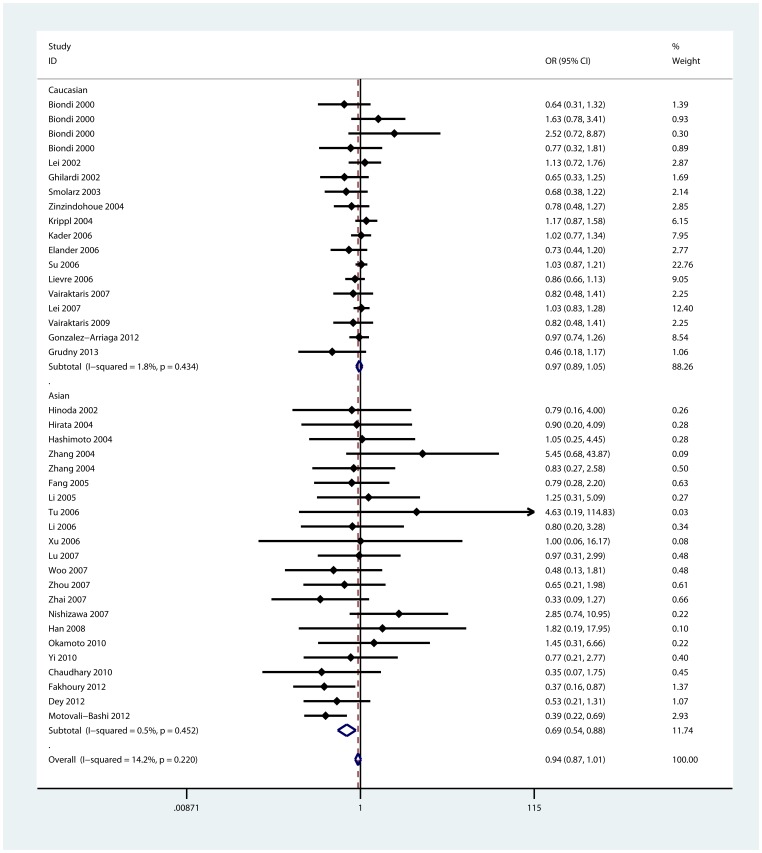
Forest plot of dominant model for overall comparison by ethnicities (6A/6A+6A/5A vs. 5A/5A).

In the stratified analyses by ethnicities, the associations were significant in Asian population for three models: homozygote comparison (6A/6A vs. 5A/5A, OR = 0.68, 95%CI: 0.52—0.90; I^2^ = 26.7%), heterozygote comparison (6A/5A vs. 5A/5A: OR = 0.75, 95%CI: 0.58—0.98; I^2^ = 0.0%), and dominant model (6A/6A+6A/5A vs. 5A/5A: OR = 0.69, 95%CI: 0.54—0.88; I^2^ = 0.5%, Figure 3). But in Caucasian population, there were no significant associations found by this sub-group analysis. In terms of sub-group analyses by genotyping method and sample size, we found significant decreased risk of cancer in the studies using PCR-RFLP method and the studies of small sample size for three models(Table 2).

### Sensitivity Analyses and Publication Bias

**Figure 4:**
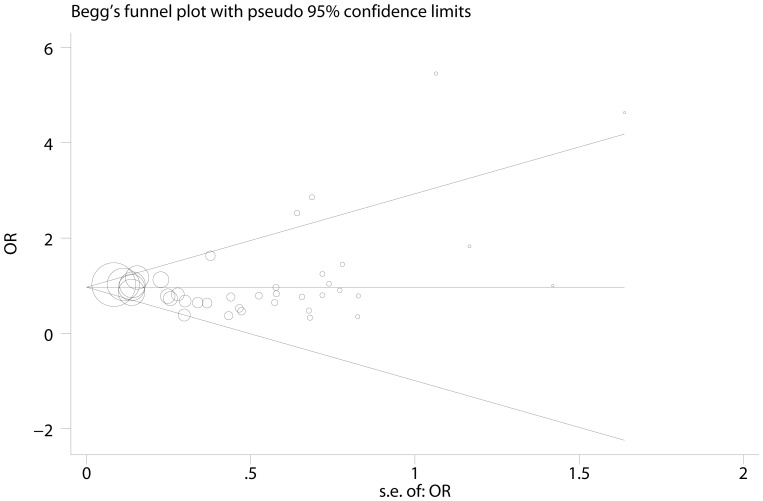
Funnel plot analysis to detect publication bias in 41 eligible studies.

One single study involved in this meta-analysis was deleted each time to reflect the influence of the individual dataset to the pooled ORs [54], and the corresponding pooled ORs were not altered (Figure S1), suggesting stability of the meta-analyses. Begg’s funnel plot and Egger’s test were performed to assess the publication bias of studies. The shape of Begg’s funnel plot was roughly symmetrical (Figure 4). The statistical results still did not show publication bias by Egger’s test (p = 0.682).

## Discussion

To our knowledge, the first meta-analysis which provided comprehensive assessment of the -1171(5A>6A) polymorphism in MMP3 promoter region and cancer risk was performed in 2010 [55]. Compared with previous meta-analysis, we updated 15 new studies(41 vs. 26). In this meta-analysis, 41 eligible studies, including 11112 cases and 11091 controls, were included and analyzed. Although numerous studies supported -1171(5A>6A) polymorphism could decrease different cancer risk, while the pooled ORs of this meta-analysis failed to confirm this association. It is worth noting that the association was significant in Asian population when compared with Caucasian population, especially in gastrointestinal cancer.

MMP3 is known to play a key role in both local invasiveness and metastasis, the latter of which involves the ability of neoplastic cells to cross the basal membrane of both the epithelium and the vascular endothelium. This is due to MMP3 can also activate gelatinase B and the collagenases and release several cell surface molecules, including E-cadherin, a known contributor to cancer development [56]. MMP3 overexpression by some cancer types is consistent with this hypothesis [57]. Apoptosis is suppressed in the presence of intact ECM basement membrane [58]. MMPs may therefore be involved in apoptosis by their ability to degrade the ECM. The insertion of an adenosine in the MMP3 gene promoter sequence halves its transcriptional activity [10]. It is conceivable that the higher transcriptional activity associated with the 5A allele may enhance tumor invasiveness. It was confirmed in this meta-analysis.

Among 41 eligible studies, carriers of the variant 6A allele were only reported with a significantly decreased cancer risk compared with those of 5A allele in gastrointestinal cancer [6], [12], [15], [27], [29], [30], [36], [48], [53]. In dominant model, there was only one study suggested the 5A allele significantly contributed to the susceptibility of lung cancer [44], but the pooled ORs failed to confirm the association in each corresponding group classified by cancer types. Furthermore, we found a significant association in head and neck cancer for homozygote comparison.

When stratified by ethnicities, we found the association between the -1171(5A>6A) polymorphism in MMP3 promoter region and cancer risk was only significant in Asians for three genetic models. The differences may be explained by genetic diversities, such as different risk factors in life styles, and various of environmental exposure [59]–[61]. Additionally, in the sub-group analysis of genotyping method, the positive result was only observed in studies using PCR-RFLP method, but not in studies using AS-PCR or Taqman method. Thus, the differences in methodology might contribute to the results in this meta-analysis.

Further analyses showed few significant results in studies of different smoking status. However, we had a contrary finding in non-smokers: the variant 6A/6A homozygote might statistically increase cancer risk compared with 6A/5A+5A/5A genotype(OR = 1.92, 95%CI: 1.25—2.96; I2 = 72.7%), which seemed to be in confliction with the previous single studies [9], [35]. The conventional view was that the genotypes containing the wild 5A allele might remarkably increase the risk of oral and lung cancer development in smokers. One possible explanation is that the effect of MMPs polymorphisms on cancer risk may be overwhelmed by the effect of cigarette smoking among smokers. Alternatively, cigarettes smoking is a major source of extracellular matrix and may induce mRNA levels of MMPs and tissue inhibitors of metalloproteases [62]. Therefore, the effect of polymorphisms affecting expression of MMP genes in smokers may depend upon the balance between MMPs and tissue inhibitors of metalloproteases [15].

Heterogeneity between studies in each model is shown in Table 2. The source of heterogeneity across studies was explored among covariables, such as cancer types, ethnicities, source of controls, sample size and genotyping method. Meta-regression results revealed that no covariables contributed to the heterogeneity across studies in the overall result. However, sub-group analyses suggested the cancer types and sample size might be the main source of heterogeneity in this meta-analysis. The studies of small sample size may contribute to a small-study effect, in which effects reported are larger, and lead to between studies variance. Publication biases were assessed by Begg’s funnel plots and their symmetries were further evaluated by Egger’s linear regression tests. The data suggested that no evident biases were observed, indicating the credibility and stability of the results.

Several limitations of this meta-analysis should be addressed. First, individual data was not available and a more precise analysis should be conducted on other covariates such as age, sex, and environmental factors. Secondly, the sample size was still relatively small for some stratified analyses. In spite of these limitations, we included 11112 cases and 11091 controls in this meta-analysis, which can increase the statistical power and strengthen the reliability of results.

In conclusion, we demonstrate that the -1171(5A>6A) polymorphism in MMP3 promoter region is not associated with overall cancer risk, but it may contribute to decreased cancer risk in Asian population when compared with Caucasian population and significantly reduce the risk of gastrointestinal cancer. To confirm these results, large scale case-control studies are required.

## Supporting Information

Figure S1 **Sensitivity Analyses.** The pooled odds ratios were calculated by omitting each data set at a time.
(TIF)

Checklist S1 **PRISMA checklist.**
(DOC)
