# Supplementary figures and images for: Association of Matrix Metalloproteinase-3 -1171(5A>6A) Polymorphism with Cancer Risk: A Meta-Analysis of 41 Studies
Source: PLoS One. 2014 Jan 29;9(1):e87562. doi: 10.1371/journal.pone.0087562 (PMC3906197; doi:10.1371/journal.pone.0087562)

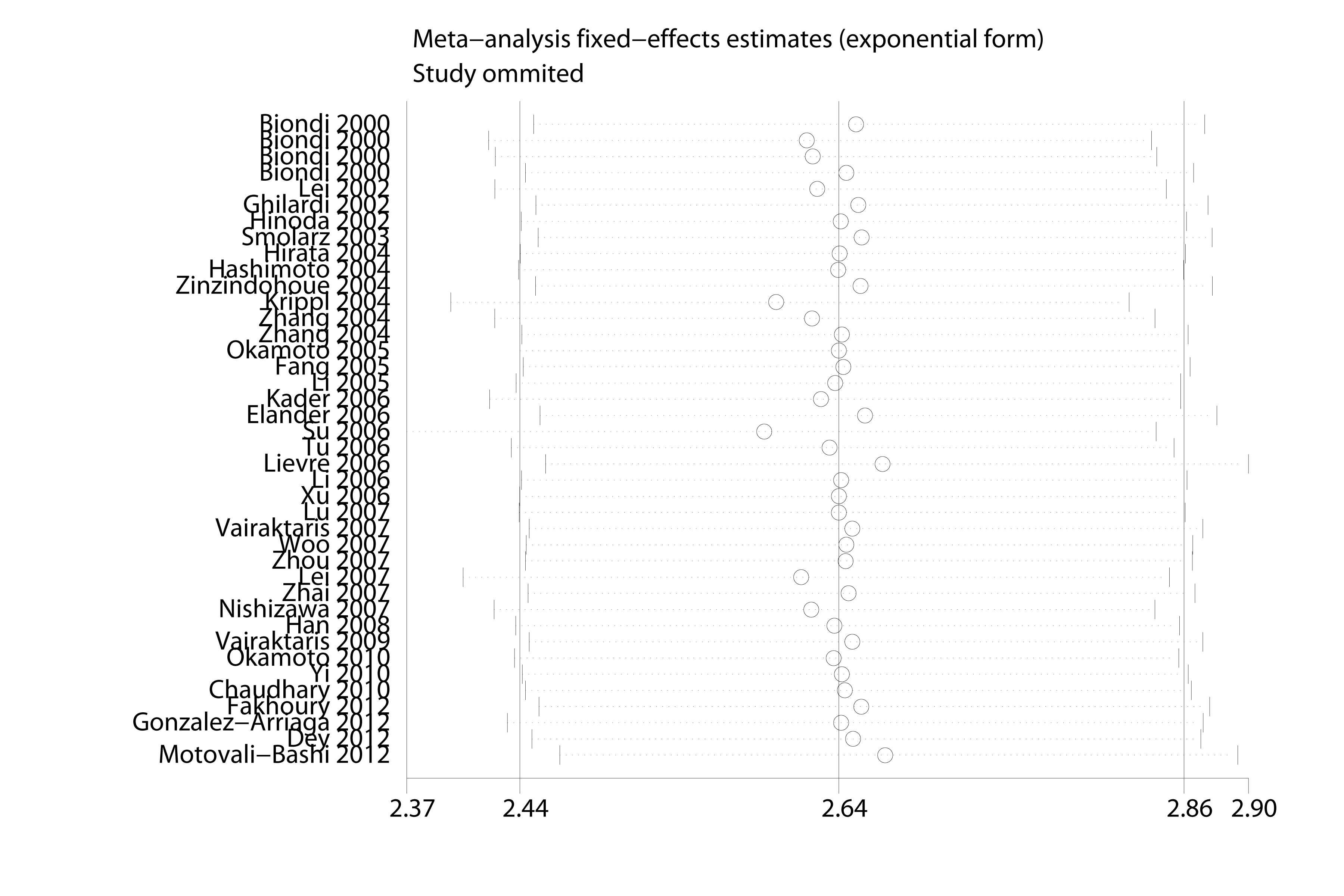

Supplement: Figure S1 — Sensitivity Analyses. The pooled odds ratios were calculated by omitting each data set at a time. (TIF) [file pone.0087562.s001.tif]
